# Overcoming Challenges Associated with Developing Industrial Prognostics and Health Management Solutions

**DOI:** 10.3390/s23084009

**Published:** 2023-04-15

**Authors:** Maxwell Toothman, Birgit Braun, Scott J. Bury, James Moyne, Dawn M. Tilbury, Yixin Ye, Kira Barton

**Affiliations:** 1 Department of Mechanical Engineering, University of Michigan, Ann Arbor, MI 48109, USA; toothman@umich.edu (M.T.);; 2 The Dow Chemical Company, Midland, MI 48674, USA; 3 Department of Robotics, University of Michigan, Ann Arbor, MI 48109, USA

**Keywords:** prognostics and health management, predictive maintenance, model design, manufacturing

## Abstract

The development of prognostics and health management solutions in the manufacturing industry has lagged behind academic advances due to a number of practical challenges. This work proposes a framework for the initial development of industrial PHM solutions that is based on the system development life cycle commonly used for software-based applications. Methodologies for completing the planning and design stages, which are critical for industrial solutions, are presented. Two challenges that are inherent to health modeling in manufacturing environments, data quality and modeling systems that experience trend-based degradation, are then identified and methods to overcome them are proposed. Additionally included is a case study documenting the development of an industrial PHM solution for a hyper compressor at a manufacturing facility operated by The Dow Chemical Company. This case study demonstrates the value of the proposed development process and provides guidelines for utilizing it in other applications.

## 1. Introduction

Prognostics and health management (PHM) research presents an opportunity for industrial manufacturers to minimize the downtime and maintenance costs associated with manufacturing processes [1]. The research field has developed rapidly in recent years, with novel methods for fault diagnosis, predictive maintenance, and maintenance scheduling being introduced regularly [2,3,4]. PHM researchers have also begun to propose general methodologies for developing and implementing PHM solutions, defined as implementations of the concepts and methods from PHM research. This includes [5], which presents a PHM life cycle that consists of design, development, and decision stages, as well as [6], which presents a methodology for designing PHM solutions that is driven by stakeholder expectations and requirements.

However, the deployment of PHM solutions in the manufacturing industry has been slow due to a number of practical challenges. Industrial manufacturing operations present a number of unique difficulties for PHM solution developers that have not been well explored, including soliciting input and support from multiple types of experts, integrating PHM solutions into existing reliability strategies, and adapting to external disturbances that impact system operation, among others [7,8]. These challenges often limit the usefulness of existing methodologies for industrial PHM solutions, but have received relatively little attention in the academic literature compared to the development of general frameworks and new modeling techniques.

The first contribution of this paper is a framework for developing industrial PHM solutions that is based on the system development life cycle (SDLC). Many of the life cycle stages have been explored in the general PHM literature, but several challenges associated with the planning and design of industrial PHM solutions have not been studied in detail. The framework includes a novel methodology for planning the scope of industrial PHM solutions, which specifies several considerations that are unique to manufacturing applications and identifies the types of experts that should be consulted during planning. A methodology for designing industrial PHM solutions builds upon existing, general PHM architectures [9,10] by proposing additional computational processes (context adaptation and output processing) that are critical in industrial applications. Additionally, two challenges that regularly afflict industrial PHM solutions, data quality and modeling degradation trends, are identified and recommendations for overcoming them are provided.

The second contribution is a collection of guidelines for implementing this framework that are based on insights from a case study PHM solution developed during a graduate student internship at The Dow Chemical Company ( The Dow Chemical Company, Midland, MI, USA). While quantitative performance results cannot be publicly disclosed (because deployment and validation of the PHM solution are currently ongoing), this case study provides a concrete example of how challenges associated with industrial PHM solutions can be overcome. The takeaways from the case study can help manufacturers to incorporate feedback from system experts into the planning and design of their PHM solutions and facilitate a structured comparison of multiple candidate solutions.

Section 2 of this paper discusses the proposed framework for developing PHM solutions and the associated challenges for industrial applications. Section 3 and Section 4 present methodologies for the planning and design stages of the development process, respectively. Section 5 describes how the framework was implemented to develop a PHM solution for an industrial hyper compressor, and Section 6 provides general insights from the project. Finally, Section 7 contains concluding remarks and recommendations for future work.

## 2. Development of Industrial PHM Solutions

The SDLC prescribes a set of stages that a system goes through from its inception to its deployment and maintenance in the field [11]. The SDLC has been used extensively in the development of software-based applications [12,13], including digital twins for manufacturing systems [8,14]. Detailed descriptions of the SDLC stages and methods for progressing through them can be found in [11,15]. While the SDLC provides a useful framework for developing a broad range of systems and applications, the decisions and considerations that should be made at each stage of the life cycle are not always clear. This section investigates two of the SDLC stages, planning and design, identifying several challenges that commonly arise when developing industrial PHM solutions.

**Figure 1:**
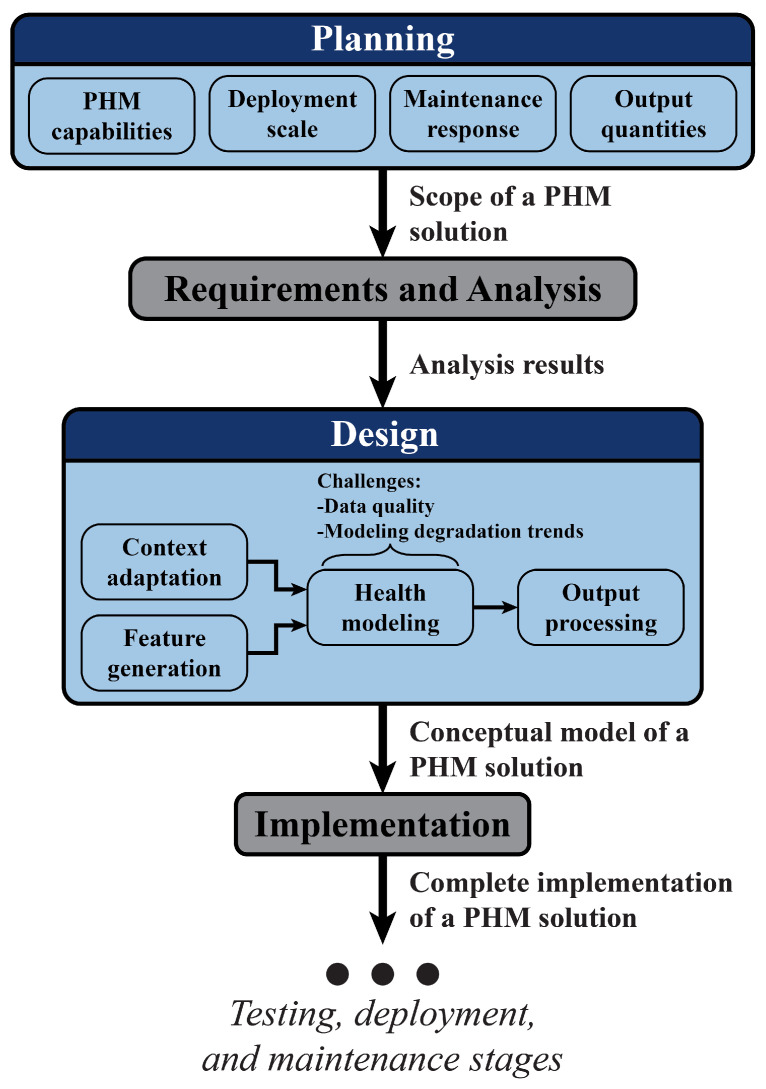
Early stages of the software design life cycle for industrial PHM solutions. This paper will discuss the planning and design stages in detail.

Figure 1 depicts the first several stages of the SDLC, which are usually completed offline based on subject matter expertise (SME) and input from end users. The transitions and output quantities in this diagram assume that the SDLC stages are completed sequentially. In reality, stages will be revisited and decisions will be revised based on insights from the subsequent testing, deployment, and maintenance stages. A number of different methodologies for transitioning through and revisiting the SDLC stages exist, including waterfall, V-shaped, and iterative methods [13,16]. This paper will focus on the first iteration of the early SDLC stages.

Planning is the first stage of the SDLC and the first of two SDLC stages that will be discussed in detail in this paper. For industrial PHM solutions, the planning stage involves defining a solution’s scope, or the role that it plays within a manufacturing operation’s broader reliability strategy. At the beginning of this stage, solution developers should identify one or more PHM capabilities that would bring value to a manufacturing operation but are currently unavailable. This decision will guide subsequent specifications concerning the solution’s deployment scale, maintenance response, and output quantities. Feedback from all system stakeholders (including reliability experts and equipment operators, among others) should be incorporated into a solution’s scope. If a PHM solution’s scope is not explicitly and thoroughly defined, its impact will suffer and the solution will not persist in the work process. Section 3 provides a methodology for defining the scope of an industrial PHM solution as well as classifications for the kinds of SMEs that should be solicited during the planning stage.

The second stage of the SDLC, requirements and analysis, uses both qualitative and quantitative analysis to determine whether an operation’s data and modeling resources are sufficient to develop a solution that satisfies the scope defined in the planning stage. The decisions and considerations that should be made during this stage are highly specific to the PHM solution being developed, so they will not be investigated in this paper. Refs. [8,14] provide a general overview of how this stage may be implemented for purpose-driven digital twin systems, which can be augmented by SMEs to carry out this analysis for individual industrial PHM solutions.

Design is the third stage of the SDLC and the second SDLC stage that will be discussed in detail in this paper. Industrial PHM solutions can combine several different models and computational processes to convert machine signals into results that are useful for minimizing system downtime. During the design stage, the structures of these processes are selected, and decisions are made about how health models will be trained. The recent literature has proposed approaches for designing PHM solutions for non-industrial applications, but industrial equipment and manufacturing environments often present additional challenges. Section 4 provides a general architecture for industrial PHM solutions that highlights processes not always considered for non-industrial applications. Two additional challenges that influence the design of industrial PHM solutions, data quality and modeling time-series degradation trends, are then discussed.

The remaining stages of the SDLC, which include implementing, testing, deploying, and maintaining an industrial PHM solution, are outside of the scope of this paper. Some stages, such as those related to implementation and deployment, will be highly specific to individual PHM solutions and manufacturing operations, while others have been examined by the general PHM literature and can be directly applied to industrial PHM solutions [14,17,18].

## 3. Planning Stage

This section first introduces four types of SME that should inform the planning stage of the SDLC for industrial PHM solutions. A methodology for completing this stage is then presented.

### 3.1. Subject Matter Expertise Classifications

Four types of SME that are necessary during the planning stage (and subsequent SDLC stages) are defined below. In practice, individual experts may possess multiple types of SME, and there may be individuals possessing “broad SME”, which overlaps with all of the classifications presented here. Those individuals should also be consulted during the planning stage to supplement the knowledge of more specialized experts.

***Business expertise***: Knowledge of the financial aspects of a manufacturing operation. Business experts have an understanding of the production demands that apply to a manufacturing operation and the financial impacts of disruptions. An understanding of the costs (absolute or relative) of false negatives and false positives from a prospective PHM solution is also necessary.
***Reliability expertise***: Knowledge of a manufacturing operation’s current reliability strategy. Reliability experts are aware of the initiatives that are currently in place to ensure that a manufacturing operation remains online, including scheduled preventative maintenance procedures and protocols for administering reactive maintenance.
***Equipment expertise***: Knowledge of the machine(s) that a prospective PHM solution will monitor. Equipment experts have an understanding of the mechanical and electrical components that make up the machine and an awareness of its potential faults and failure modes.
***Data expertise***: Knowledge of a manufacturing operation’s data collection capabilities. Data experts are aware of the signals that are available to be used in a PHM solution and understand how system health problems can manifest themselves in these signals. They are also familiar with the data reporting and storage infrastructure that will be used to implement the PHM solution and can provide insight into how data collection and processing can be improved to better support PHM solutions.

### 3.2. Planning Methodology

**Figure 2:**
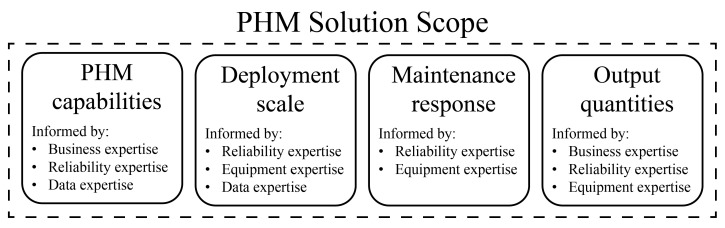
Specifications that constitute the scope of an industrial PHM solution and the subject matter expertise that should inform them.

For industrial PHM solutions, the planning stage of the SDLC consists of defining four specifications that describe the solution’s role in a manufacturing operation, referred to as the scope of a PHM solution. These specifications and the SME that should inform them are depicted in Figure 2. Each specification is described below in a suggested definition order, but the appropriate order for individual solutions may vary.

The definition of a PHM solution’s scope should begin by specifying one or more high-value problems that exist within an operation’s current reliability strategy. Based on this decision, one or more modeling and analysis tasks can be selected to address these problems. The academic research community has identified many PHM-related tasks [19,20], which will be referred to as PHM capabilities. Several PHM capabilities are defined in [21,22], including detecting ongoing equipment degradation, diagnosing the root cause of degradation, and predicting when maintenance will be necessary. General anomaly detection and repair quality assessment are other examples of PHM capabilities that may be enabled. If multiple capabilities are identified as potentially useful, they may all be included in the scope of a prospective PHM solution. Validation results obtained later in the development process will dictate which capabilities can actually be delivered. Reliability expertise is necessary here to estimate the benefits of each PHM capability in terms of system uptime. Business expertise is also necessary to weigh the financial impacts of these benefits against the cost of developing a PHM solution that enables the desired capabilities. Finally, data expertise is needed to determine if a PHM capability can be enabled and maintained with the current data system or if the data system can be improved to better support a capability.

A PHM solution’s scope should also specify the scale at which the solution will be deployed. This includes basic decisions about which piece of equipment or equipment subsystem to monitor, which should be motivated by pre-existing reliability shortcomings. Deployment scale specifications may also identify events that will trigger the activation or deactivation of a solution. For example, it may be appropriate to deactivate a PHM solution around startup and shutdown events because machine signals can be erratic during these periods. If several similar machines are part of an equipment fleet, then a decision should be made about how many of these machines will be monitored by the PHM solution. Reliability and equipment experts should use their understanding of the likelihood of health problems throughout a machine’s lifetime to inform these decisions. Data experts should also weigh in on the sensing capabilities across equipment fleets, which may influence the scale at which a solution can be deployed.

A set of maintenance actions that may be taken based on the outputs of a PHM solution should be specified next. Potential actions range from intensifying the manual supervision of a system to immediately shutting down a system for repair. Maintenance actions can be triggered automatically based on the outputs of a PHM solution, or can be the result of a negotiation between a PHM solution and an equipment operator. If the latter is preferred, the negotiation questions and a set of potential actions should be specified. Reliability expertise is necessary here to define how a prospective PHM solution can fit into an operation’s existing reliability strategy. A key aspect of this specification is the amount of time that is necessary to take these actions. Equipment expertise should also be solicited to ensure that the specified actions are feasible and compatible with the allotted time frame.

Finally, a set of desired output quantities from the prospective PHM solution and the minimum levels of accuracy that would deliver a net benefit to an operation should be specified. PHM solutions designed for fault or degradation detection may provide discrete-state classifications as outputs, while solutions designed for fault prediction may provide continuous remaining useful life (RUL) predictions. Any desired measures of uncertainty, such as state probability estimates or confidence intervals, should also be specified here. Input from equipment experts is necessary to select output quantities that will allow the actions specified above to be taken. Business and reliability experts should also use their understanding of an operation’s downtime and maintenance costs to specify minimum accuracy rates for a prospective solution.

## 4. Design Stage

This section is focused on the design of industrial PHM solutions. A general architecture for industrial PHM solutions is first proposed. The section then introduces two challenges that commonly arise in manufacturing environments: data quality and modeling time-series degradation trends. Several methods to adapt the design of equipment health models to overcome these challenges are discussed.

### 4.1. PHM Solution Architecture

**Figure 3:**
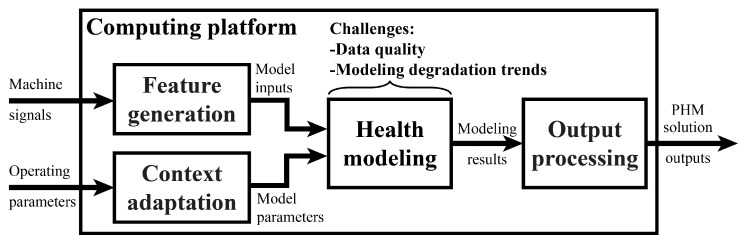
PHM solution architecture with SME foundations.

Figure 3 depicts a general architecture for industrial PHM solutions that includes a set of computational processes and a flow of information between them. A foundational aspect of the architecture is a computing platform that supports these processes and aggregates input data streams. The choice of an appropriate computing platform will be strongly influenced by a company’s information technology (IT) and data security policies. For any application, establishing a reliable connection from data acquisition devices and historical databases to the chosen computing platform is essential.

The computational processes described below represent implementation choices that developers must make when designing a PHM solution. When developers identify multiple options for implementing a computational process, different candidate solutions can be designed in parallel and represented based on this architecture. Each process is described below in their runtime order, or the order in which they are executed when a PHM solution is actively monitoring the real-time health of a system. It is not necessary to design a solution in this order, though, and processes may be revised multiple times based on testing results.

A feature generation process receives signal measurements from a machine and computes a set of model input features from those measurements. Common feature generation processes include extracting averages from windows of signal measurements or performing Fourier transforms on vibration data to extract frequency domain features. When many different signals are available from a system, equipment experts can greatly simplify the feature generation process by identifying a subset of machine signals that contain useful information about the health of a system. Data experts should also be relied upon here to translate qualitative descriptions of unhealthy behavior provided by equipment experts into quantitative signal features that can be used as model inputs.

Context adaptation can be executed in parallel with feature generation during online monitoring. This architecture defines the system context as the operating parameters and environmental factors that change over a system’s lifetime (either intentionally or as a side effect of degradation) and impact the machine signals monitored by a PHM solution (and thus the behavior being detected or predicted by the PHM solution). Rotating speed and product type are two examples of parameters that commonly impact system behavior. A context adaptation process tracks a set of context states and maps these state values to modeling parameter values that dictate how a system is analyzed in its current context [22]. While a PHM solution may forgo context adaptation by keeping modeling parameters constant throughout a system’s lifetime, it is often beneficial to use context adaptation to translate system health models or switch between multiple models based on system context. Equipment experts often have an understanding of the parameters that vary throughout a system’s lifetime and how these changes impact system behavior. This expertise should be used to identify a set of context state candidates that are considered for inclusion in a solution’s context adaptation process.

The health modeling process uses the outputs from feature generation and context adaptation to produce modeling results that describe system health in some way. The recent literature has proposed a diverse set of health modeling approaches, but only a small number of these may be appropriate based on the scope of the prospective PHM solution. Solution developers should carefully consider two challenges that commonly afflict industrial PHM solutions: data quality and modeling time-series degradation trends. While academic research commonly uses data collected from defective equipment or run-to-failure tests, it is usually not feasible to collect these data from manufacturing equipment that must be kept online. Historical measurements collected during online operation are often the only available datasets and require careful, SME-informed pre-processing to be used for model training. Additionally, equipment failures may be rare events, which makes these datasets unbalanced and presents difficulties for training many of the machine-learning-based models proposed in the recent literature. Another challenge for PHM developers is equipment degradation, which is characterized by time-series trends in machine signals (potentially beginning from varying baseline values). When this is a possibility, equipment health models should be designed to consider the histories of recent observations when estimating current and future health states. Methods that neglect system history when making health state estimates are either limited in their ability to detect early-stage degradation, such as univariate signal limit monitoring, or hyper-specific to a single application, which limits their robustness to disturbances. Section 4.2 and Section 4.3 discuss methods that can be used to overcome both of these challenges when designing industrial PHM solutions.

Finally, output processing consists of any computations to format or synthesize modeling results into solution outputs that are delivered to end users. This may involve aggregating or merging the outputs of multiple health models, or appending estimates of uncertainty to modeling results. Output processing may not be necessary for all applications, but can be used in some cases to convert modeling results into the output quantities specified by a solution’s scope. Both equipment and reliability experts should be consulted when selecting an output processing approach to ensure that the implications of solution outputs are clear and actionable for the solution’s end users.

### 4.2. Data Quality

#### 4.2.1. Pre-Processing Historical Data

Early in the development of a PHM solution, decisions must be made about the source of data that will be used to train the health models within a PHM solution. In many industrial applications, databases of machine signal measurements from periods of online operation, referred to as historical datasets, are the only available source. In their raw form, though, these datasets are not well suited for model training. A method for filtering and labeling these historical datasets is presented here.

The method begins by identifying the following events in a historical dataset. Equipment maintenance logs and historical equipment state tag values can help accomplish this.

Fault: An occurrence associated with an unwanted situation within an manufacturing system that must be resolved through maintenance

Shutdownforrepair: Transition of a system from an online state to an offline state for the purpose of conducting a repair procedure

Startupafterrepair: Transition of a system from an offline state to online state after a repair procedure has been completed

**Figure 4:**
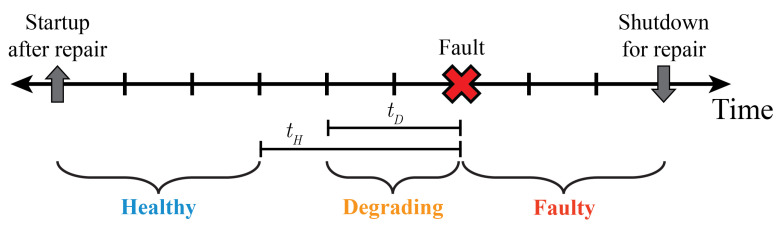
Historical data labeling convention.

Figure 4 shows an example timeline for a historical dataset with these events labeled. Input from equipment and reliability experts should be used to specify an assumed degradation length ($t_{D}$) and a healthy buffer length ($t_{H}$) for the system and fault being monitored. The value of $t_{D}$ defines the maximum time prior to a system fault that a system can reasonably be assumed to be Degrading. The value of $t_{H}$ defines the minimum time prior to a system fault that a system can reasonably be assumed to be Healthy. Periods of historical data can then be labeled as Healthy, Degrading, or Faulty based on these parameters, as shown in Figure 4. The labeled datasets can be used to train models for different PHM capabilities, including degradation detection, fault detection, and fault prediction.

#### 4.2.2. Combined Training Datasets

PHM solution developers may also be limited by the amount of data that are available for model training. Industrial equipment often undergoes shifts in operating conditions, such as maintenance events involving mechanical design changes, that change the distribution of machine signals and render older historical data unsuitable for model training. Additionally, historical datasets usually suffer from class imbalance, characterized by a small amount of unhealthy measurements relative to healthy measurements. This can be the result of rare Fault events or the practice of carrying out preventative maintenance procedures before system degradation begins. Limited training data limits both the types of health models that can be implemented in a PHM solution and the accuracy of the solution.

To overcome data sparsity in individual machines or contexts, a context adaptation approach can combine datasets from related machines or contexts. In practice, this can mean combining data from identical machines operating in the same plant, data from similar machines operating in different environments, or even data from the same machine from different periods of time (before and after machine overhauls, for example). Previous research has proposed several methods for applying knowledge learned in one domain to other domains, a transfer learning approach known as domain adaptation [23,24]. The developers of an industrial PHM solution may choose to implement any of these methods, which span a wide range of theoretical and computational complexities. The recent literature has also proposed metrics for quantifying the similarity of datasets from different machines or contexts. Maximum mean discrepancy is often used to estimate the difference between datasets and both transfer component analysis and linear discriminant analysis are designed to identify low-order transformations that align datasets from different domains [25,26]. These analysis methods can help avoid decreases in model accuracy that result from combining datasets with drastically different distributions.

An initial foray into domain adaptation can be realized by simply normalizing datasets from separate contexts before combining them into a single model training dataset. This entails defining a set of *k* context states that are each associated with a system or operating condition that is considered distinct from other context states. Mean and standard deviation vectors $\mu_{i},\sigma_{i}$ are calculated for each context state, based on the data collected under those conditions, where 1≤i≤k. The data associated with each context state are then normalized based on these mean and standard deviation vectors before combining all data into a combined dataset that is used for model training. If a PHM solution is developed with this approach, the context-specific mean and standard deviation vectors must be retained to normalize new measurements before providing them as inputs to the trained models.

### 4.3. Modeling Time-Series Degradation Trends

Another challenge that should be accounted for when designing an industrial PHM solution is the possibility of time-series degradation trends in manufacturing equipment. Recognizing gradual degrading behavior in industrial equipment is often crucial for triggering low-cost maintenance procedures and avoiding downtime. Many complex equipment degradation processes also experience multiple discrete degradation stages that are characterized by different machine signal behaviors. To account for these phenomena, health models can maintain estimates about the current state of equipment health that are informed by historical measurements, an approach that will be referred to as state-based health modeling.

General path modeling is an example of a state-based modeling approach that identifies gradual degradation trends in machine signals and extrapolates this behavior to make failure predictions [27,28]. Certain general path models have a small number of stochastic parameters that can be re-estimated according to a Bayesian update strategy when new signal measurements are made. The resulting failure predictions are then probabilistic windows that correspond to a user-specified level of certainty. When equipment faults are rare and training data are limited, these low-complexity models can be more practical to train and deploy than deep learning models.

A survey of other model-based methods for detecting and diagnosing degradation can be found in [29]. These include strategies that represent equipment dynamics and degradation trends using state-space models or differential equations. Observer-based methods can then be used to estimate health-related model parameters based on system outputs [30,31] or compute residuals between expected and actual system outputs that indicate degradation [32,33].

In cases where developers would like to capture multiple discrete degradation stages in a health model, hidden Markov models (HMMs) are a useful modeling tool [34,35]. The unobservable states in an HMM can be used to represent discrete system health stages, including a Healthy state characterized by steady-state behavior in machine signals, and one or more unhealthy states, such as Degrading or Faulty states, that are characterized by dynamic signal behavior or undesirable signal levels. A Degrading state may also be divided into multiple sub-states to represent different physical degradation mechanisms that are known to precede system faults. In an HMM, each of the unobservable states are associated with an observation probability distribution that describes the likelihood of certain observable features. For PHM solutions, these observable features may be the values of machine signals or trend-based features extracted from windows of machine signals [36]. The Viterbi algorithm can then be used to compute the most likely state history of a system based on historical observations.

HMM state estimation methods can also be used to incorporate state-based modeling into pre-existing health state classifiers (when a model’s historical accuracy rates are known). To achieve this, a model’s classification results are treated as observable features. In an HMM, each state’s observation probability distribution defines the probability of making observation *o* at time *t* when the system is in state $S_{i}$. This can be expressed as

(1) $$b_{i}\left(o\right)=P\left(o\;\mathrm{at}\;t|q_{t}=S_{i}\right)$$

where $B=b_{i}\left(o\right)$ is the observation probability distribution of state $S_{i}$ and $q_{t}$ is the true state of the system at time *t*. A classifier’s accuracy rates, often formatted as a confusion matrix, express the same likelihoods. Observations at each time *t* are the predicted state classification ($\hat{q_{t}}$), and $b_{i}\left(\hat{q_{t}}\right)$ is the likelihood of receiving a $\hat{q_{t}}$ classification when the true system state is $S_{i}$. A classifier’s true-positive rate (TPR) and false-positive rate (FPR) define the observation probability distributions in each state. The Viterbi algorithm can then be used to make health state estimates based on a history of classification results.

In some applications, Markov models of equipment health can enable predictive capabilities. Hidden semi-Markov models are used most often for fault prediction or RUL estimation [37]. These models allow state transition probabilities to change over time to reflect system deterioration. The time remaining until a system enters a failure state, commonly referred to as remaining useful life (RUL), can then be estimated using methods described in [38,39].

## 5. Case Study PHM Solution Development

This section describes the process of developing a PHM solution during a graduate student internship at The Dow Chemical Company. The details presented here have been reviewed by the company and approved for external release. Each of the SDLC stages shown in Figure 1 were used to develop four candidate PHM solutions that are currently being tested before deployment. These solutions and the process of developing them are presented here.

### 5.1. Planning

The planning stage of the development process for this PHM solution involved defining the solution’s scope based on a pre-existing reliability problem at a low-density polyethylene (LDPE) manufacturing operation. In recent years, the operation has been plagued by frequent faults in an industrial hyper compressor that result in emergency shutdowns and lost production. Business and reliability experts first determined that the manufacturing operation would benefit from a PHM solution that provides degradation detection capabilities. Such a solution would provide warnings of impending faults in the machine.

Reliability, equipment, and data experts agreed that the system should be deployed to monitor the health of the hyper compressor during all periods of online operation. Additionally, because the hyper compressor consists of eight cylinders that operate in parallel and are all susceptible to faults, all cylinder subsystems were included in the PHM solution’s scope. Equipment experts were then consulted to define how a PHM solution could be integrated into the plant’s existing reliability strategy. They concluded that, given an indication of an impending fault in the hyper compressor, equipment and reliability experts would first attempt to resolve the fault without a shutdown by manipulating the operating parameters of the machine. If this is not possible or unsuccessful, equipment maintenance would be scheduled at a convenient time in the near future.

Finally, reliability and equipment experts specified a set of desired PHM solution outputs. An important decision was made here to monitor the health of each hyper compressor cylinder individually, rather than monitoring the overall health of the machine. The most desirable output of the prospective PHM solution is indications of degradation in each cylinder in the form of a discrete health state from the set {Healthy,Degrading}. Another desirable output quantity from a prospective PHM solution is a set of signal identifiers that accompany Degrading health state classifications to indicate the machine signals that are deviating from the expected behavior. This information may help equipment experts to identify the root cause of degradation and determine if it can be resolved without a shutdown. Experts also concluded that the PHM solution would need to provide warnings of ongoing degradation approximately 2 weeks before system fault to have sufficient time to investigate the problem and schedule system maintenance. Because the PHM solution is meant to serve a purely advisory role in the operation’s reliability strategy, no minimum accuracy rates were imposed on the solution at this stage.

### 5.2. Requirements and Analysis

At this stage in the development process, experts conducted an analysis to gauge the feasibility of the proposed PHM solution. Several years ago, the hyper compressor was equipped with several vibration sensors on each cylinder to facilitate the detection of faults and degradation. Other standard operating sensors, such as inlet and outlet gas properties, are also available to be used as inputs to a PHM solution. Additionally, the manufacturing operation has a historical dataset of these measurements dating back several years along with equipment failure dates that can be used for model training. Experts agreed that these sensing capabilities and training datasets would be adequate to develop a PHM solution to enable degradation detection.

### 5.3. Design

**Figure 5:**
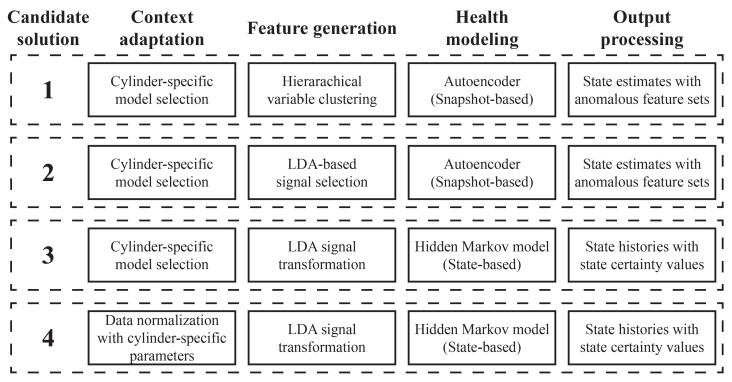
Architectures of four candidate PHM solutions.

The design stage of the PHM development process resulted in four PHM solution candidates, each with a different set of implementations for the four computational processes discussed in Section 4.1. The architectures of the solution candidates are depicted in Figure 5 and each implementation approach is described below.

#### 5.3.1. Context Adaptation

Different context adaptation approaches were developed to satisfy requests for cylinder-specific health state estimates. The first approach, used in Solutions 1–3, involves building cylinder-specific models based on data from each cylinder individually. A cylinder identifier, which can be considered a context state, accompanies new measurements and dictates which model is used to make health state estimates. An alternate approach, used in Solution 4, normalizes historical data from each cylinder and combines them to form a combined training dataset, as described in Section 4.2. With this approach, cylinder identifiers dictate the parameters used to normalize new measurements before providing these values as inputs to a single health model that makes health estimates for all cylinders.

#### 5.3.2. Feature Generation

The LDPE manufacturing operation collects a large amount of data from various pieces of equipment and subsystems, so sensor selection and feature generation is a critical aspect of the PHM solution. With the help of equipment and data experts, a subset of the operation’s signals were identified as potentially relevant for monitoring the health of the hyper compressor. The signals in this subset were used as inputs to a set of feature generation approaches that were proposed and tested during the development process.

The feature generation approach in Solution 1, known as hierarchical variable clustering (HVC), uses PCA to form clusters of highly correlated signals and select a signal representative from each cluster [40]. Historical data from the hyper compressor were labeled according to the process described in Section 4.2, and data from periods of Healthy and Degrading operation were combined to form a training dataset for the algorithm. While data from Degrading periods were used here, the HVC algorithm can be used to generate features from Healthy data alone if data sparsity is a concern for an application. The outcome of this approach is a reduced number of signals that capture the variability of the training dataset and can be used as health model input features.

The feature generation approaches in Solutions 2–4 are based on the linear discriminant analysis (LDA) method described in [26]. LDA seeks to derive a linear transformation that minimizes the distance between data points with identical classifications and maximizes the distance between data points with different classifications. Conventional LDA is used in Solutions 3 and 4 to determine a set of components that map the original machine signals to smaller, transformed input features. Historical data with Healthy and Degrading classifications were used to determine these components. However, it can be difficult to interpret the meanings and implications of these transformed features. Solution 2 uses a modified feature generation approach that seeks to identify model input features that exhibit the greatest difference between Healthy and Degrading periods. The approach first ranks the original machine signals based on the magnitude of their coefficients in the first LDA component. The *d* highest-ranking machine signals are then selected as health model input features. The parameter *d* is set to be equal to the number of variable clusters computed for Solution 1, so that the same autoencoder model can be used in both candidate solutions.

#### 5.3.3. Health Modeling

Candidate solutions 1 and 2 use autoencoder health models to make health state classifications for each cylinder. Autoencoders are a form of neural network that seek to compress and then reconstruct their input features [41]. Autoencoders are well suited for applications where equipment faults are rare because they can be trained with exclusively Healthy historical data, which are readily available from most manufacturing equipment. Significant deviations between the original features and the reconstructed features indicate anomalous behavior in the system being monitored. Health state classifications are obtained here by checking whether the root-mean-square error (RMSE) between original and reconstructed features exceeds a pre-defined threshold. If so, the system is classified as Degrading. Otherwise, the system is classified as Healthy. The structure of the autoencoder networks used in these solutions was optimized to provide robust representations of the input space during normal operating conditions. Because the autoencoder implemented here uses only the most recent measurements from the hyper compressor to make health state estimates, this modeling approach is denoted as “snapshot-based” in Figure 5.

**Figure 6:**
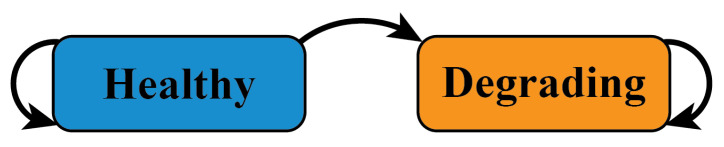
Hidden Markov model statechart.

Solutions 3 and 4 use HMMs to make health state classifications. The statechart shown in Figure 6 depicts the HMM’s unobservable states and possible transitions. The observation probability distributions for each unobservable state are estimated by fitting a Gaussian distribution to historical data from Healthy and Degrading periods. In Solution 3, different observation probability distributions are estimated for each cylinder using data from that cylinder only. In Solution 4, normalized data from all cylinders are combined into larger Healthy and Degrading datasets that are used to estimate the HMM’s observation probability distribution. Identical copies of the HMM are then deployed to monitor each cylinder. As described in Section 4.3, the Viterbi algorithm is used to estimate the health state history of each cylinder whenever new signal measurements are collected. Because the health state estimates provided by a hidden Markov model are informed by a recent history of hyper compressor measurements, this modeling is denoted as “state-based” in Figure 5.

#### 5.3.4. Output Processing

All solutions developed for this application provide the most important output quantity: state estimates from the set {Healthy,Degrading} describing the current health state of each hyper compressor cylinder. However, each solution carries out computations to provide additional outputs that add context to the health state estimates. It is critical to consider the full set of outputs that each solution provides, alongside measures of testing performance, when deciding which candidate solution to deploy.

For Solutions 1 and 2, which use an autoencoder modeling structure, health state estimates are determined based on the combined RMSE of all input features. However, the solution’s input features can also be ranked by comparing feature-specific error values with one another. This information augments Degrading state estimates with an ordered set of input features that deviate most significantly from the expected behavior. The highest-ranking features can inform efforts to investigate and resolve system degradation.

Because of their feature generation implementations, Solutions 3 and 4 are not able to provide sets of anomalous input features, but can provide probabilistic state certainty values and retroactive estimates of previous health states. These quantities are computed as part of the Viterbi state estimation algorithm, so no further additional computations are necessary to derive them. State certainty values allow system and reliability experts to calibrate their maintenance response based on the confidence that a solution has in its health state estimates. For example, one may choose to merely monitor a system more closely when certainty in a Degrading state estimate is low, or to immediately schedule a planned shutdown when confidence in a Degrading state estimate is high. The solutions’ ability to revise previous health state estimates may be useful when appropriate maintenance actions are dependent on the time since system degradation began. Measurements that were once considered Healthy may be revised to Degrading based on subsequent measurements, for instance, allowing experts to trigger a more aggressive maintenance response.

## 6. Case Study Insights

This section discusses general takeaways from the early stages of developing the case study PHM solution using the methods presented in Section 3 and Section 4, as well as guidelines for applying these methods in other applications.

### 6.1. Planning

The methodology detailed in Section 3 guided the planning stage and organized the input received from the PHM solution’s various stakeholders. An important piece of input solicited during this process was requests from reliability and maintenance experts for modeling outputs that could be used to investigate the root cause of system degradation. This information influenced the health modelling and output processing approaches in Solutions 1 and 2. Another impactful decision made during the scope definition process was the 2-week detection requirement. The assumed degradation length ($t_{D}$) was set to 2 weeks because of this requirement, which had significant ramifications for the size of model training datasets and model performance. It is critical to specify this value early in the model development process to ensure a PHM solution’s long-term effectiveness. When developers impose a similar detection requirement on their solution, a feasibility study should be conducted during the requirements and analysis stages to determine whether the available machine signals exhibit anomalous behavior prior to the minimum detection time.

### 6.2. Design

The PHM solution architecture and computational processes introduced in Section 4 helped to standardize the development and comparison of candidate PHM solutions. This made it easier to identify when approaches can be combined, such as Solution 1 which uses an autoencoder with input features generated from hierarchical variable clustering, or substituted, such as Solutions 3 and 4 which use different context adaptation approaches. The proposed architecture also draws attention to context adaptation and output processing decisions, which are commonly overlooked in industrial PHM solutions. Decisions about which context states are incorporated into a solution and what output quantities a solution delivers have a considerable impact on its accuracy and value for end users.

### 6.3. Data Quality

A primary takeaway from implementing the strategies proposed in Section 4.2 for filtering and labelling historical training data is the need for high-quality documentation on machine faults and shutdowns. This information is necessary to identify the historical Fault events and the period of system degradation. If the machine conditions that motivate a machine shutdown and the duration of the problem are not recorded in sufficient detail, it is impossible to pre-process historical data for model training.

Like most industrial applications, there are a limited number of historical degrading periods for each of the hyper compressor cylinders. PHM Solution 4 was designed to address this limitation by training a single health model with historical data from all cylinders. Early testing shows that the approach improved degradation detection capabilities in all cylinders, most significantly in cylinders with relatively few periods of degradation in their histories. This result suggests that data normalization can be an effective approach for identifying degradation trends that are shared between machines or machine subsystems and boosting the accuracy of system models with limited training data.

### 6.4. Modeling Time-Series Degradation Trends

HMMs were utilized in Solutions 3 and 4 to implement a state-based analysis of hyper compressor system health. Compared to the autoencoder solutions, the HMM solutions exhibited fewer false positives in preliminary tests, which increased the overall accuracy rates. Their ability to consider a time series history of recent measurements when making state estimates proved valuable in this application, especially when brief process disruptions introduce variability into signal measurements.

The performance gains, however, come with added model training work and reductions in model transparency. HMM parameters, including observation probability distributions and state transition probabilities, must be estimated prior to model deployment. Strategies for estimating HMM parameters can be found in [34,36], but they are not as well defined or as automated as parameter tuning methods for other data-driven model types. Additionally, Solutions 3 and 4 use a small number of transformed features as model inputs to reduce computational complexity. Because the features are linear combinations of a large number of machine signals, an operator’s ability to identify the root cause of degradation based on feature trends may be diminished. In some applications, this limitation can outweigh the benefits to model accuracy that an HMM achieves.

## 7. Conclusions

The recent academic literature includes a wide variety of PHM-related modeling approaches, but the practical challenges associated with deploying them in industrial environments are not always considered. This work seeks to promote an awareness of the barriers between academic PHM research and industrial PHM solutions and propose tools for overcoming them. The authors believe that the proposed framework will be valuable to manufacturers as they integrate PHM solutions into their reliability strategies.

An area that the authors did not explore in detail is how knowledge about the operation of a system should influence the design of PHM solutions. The case study discussed here was limited by a fixed historical database that could be used for model training, and by limited prior knowledge of how the operation of the hyper compressor changes during degradation. Future research may expand on this work by developing formal processes for SMEs to describe system health stages or various types of system anomalies. The observability of these stages and anomalies can then inform a solution’s approach to feature generation and health modeling.

As PHM concepts become more widely accepted in industry, additional implementation challenges will inevitably arise. These will likely include the need to maintain and adapt PHM solutions throughout their lifetime, a capability that academic research is just starting to explore [8]. Related difficulties will arise when PHM solutions are scaled up to monitor the health of manufacturing equipment fleets. Methods for tuning modeling resources to new contexts and environments must be developed to accommodate this. Regardless of how these and other challenges are addressed, it will be essential to consider the constraints of industrial operations and to design future PHM tools accordingly.

## Data Availability

The dataset used in this research is confidential and not approved for public release.
